# Monitoring of the Main Reasons for Early Abandonment of Breastfeeding during the First Six Months of Life: A Secondary Analysis

**DOI:** 10.3390/nursrep14030144

**Published:** 2024-08-09

**Authors:** María Jesús Valero-Chillerón, Francisco Javier Soriano-Vidal, Desirée Mena-Tudela, Águeda Cervera-Gasch, Rafael Vila-Candel, Irene Llagostera-Reverter, Laura Andreu-Pejó, Víctor Ortíz-Mallasén, Víctor Manuel González-Chordá

**Affiliations:** 1 Nursing Research Group (GIENF Code 241), Nursing Department, Universitat Jaume I, 12071 Castellόn, Spain; 2 Joint Research Unit PECAWOL (Perinatal Care and Women’s Health) FISABIO-UJI, 46020 Valencia, Spain; 3 Foundation for the Promotion of Health and Biomedical Research in the Valencian Region (FISABIO-SP), 46020 Valencia, Spain; 4 Department of Nursing, Universitat de València, 46010 Valencia, Spain; 5 Department of Obstetrics and Gynaecology, Hospital Lluis Alcanyis, 46800 Xàtiva, Spain; 6 Feminist Institute, Universitat Jaume I, 12071 Castellón, Spain; 7 La Ribera Primary Health Department, 46600 Alzira, Spain; 8 Faculty of Health Sciences, Universidad Internacional de Valencia (VIU), 46002 Valencia, Spain; 9 Nursing and Healthcare Research Unit (INVESTÉN-ISCIII), Institute of Health Carlos III, 28029 Madrid, Spain

**Keywords:** breastfeeding, exclusive breastfeeding, early weaning, exclusive breastfeeding cessation, breastfeeding abandonment, nursing, women

## Abstract

The rate of six-month-old infants exclusively breastfed in Spain remains below the recommended rate. This study aimed to explore in detail the evolution of feeding during the first six months of life of a group of newborns, as well as to identify the reasons reported by the mothers for feeding change. A secondary analysis of two prospective longitudinal observational studies was conducted. In both studies, women participants, during the clinical puerperium, opted for exclusive breastfeeding for their newborns. The participants were followed up during the infants’ first six months. A sample size of 314 participants was obtained, of which 77.1% (*n* = 232) were of Spanish origin, and 51% (*n* = 160) were primiparous. The prevalence of exclusive breastfeeding at six months was 55.4% (*n* = 174). During the first four months of life, the main reason for early abandonment of breastfeeding was the perception of insufficient milk production. After the fourth month, the predominant reason was starting work. Statistically significant differences were observed between the reasons for giving up and the total weeks of exclusive breastfeeding (*p* < 0.001) and total weeks of breastfeeding (*p* = 0.002). Early weaning from breastfeeding is a multifactorial phenomenon. However, depending on the moment cessation occurs, some reasons predominate over others and, in many cases, can be prevented. These results indicate the need to continue investing efforts to promote and protect breastfeeding in Spain.

## 1. Introduction

Breast milk is the optimal food for infants’ proper growth and development. It provides all the energy and nutrients necessary during the first six months of an infant’s life and continues to provide half or more of the nutritional requirements up to the first year of life and up to a third during the second year [1]. Breastfeeding, in general, especially natural breastfeeding offered directly from the mother’s breast, presents both short- and long-term benefits for infants, mothers, society, and the environment [2].

International organisations, such as the World Health Organization (WHO) and the United Nations Children’s Fund (UNICEF), along with various scientific societies, recommend exclusive breastfeeding during the first six months of an infant’s life and from then on to continue breastfeeding with complementary foods [3,4,5].

However, the figures for exclusive breastfeeding are below the target set by the WHO for 2025 to ensure that at least 50% of infants are exclusively breastfed [6]. In fact, according to the 2017 National Health Survey in Spain, only 39% of infants under six months were breastfed [7].

The abandonment of breastfeeding is a multifactorial and multilevel phenomenon. The abandonment of breastfeeding is a multifactorial phenomenon in which various determinants can intervene, such as sociocultural and socioeconomic factors. In the same way, it is also a multilevel phenomenon since individual factors of the mother or baby may intervene, as well as the mother’s interaction with health services, the family, or the work environment [8]. In Spain, various studies over the years have addressed various reasons for early abandonment of breastfeeding [9,10,11,12]. The objective of this study was to explore in detail the evolution of feeding during the first six months of life in a group of newborns from the Valencian Community who, upon hospital discharge, were exclusively breastfed. This study also aimed to identify the main reasons reported by mothers that lead to early weaning from breastfeeding.

## 2. Materials and Methods

### 2.1. Study Design

A secondary analysis of the data obtained from two observational, longitudinal, and prospective studies was carried out at three hospitals in the Valencian Community (Spain): The General University Hospital of Castellón (Department of Health, Castellón), the University La Ribera Hospital (Department of Health, La Ribera), and the Lluís Alcanyís Hospital of Xátiva (Department of Health, Xátiva-Ontinyent).

### 2.2. Data Collection Procedure

The recruitment of the participants took place during the clinical puerperium. Specifically, the recruitment period for the first study was from December 2018 to May 2019 (*n* = 114) [13], while that for the second study was from April 2022 to March 2023 (*n* = 200) (this study is currently in the process of being published). The two studies from which the present secondary study originated pursued two different objectives related to breastfeeding. Therefore, exclusive breastfeeding and total breastfeeding were monitored during the first six months of the babies’ lives. Likewise, in both cases, the main reason for abandoning breastfeeding was recorded if it occurred before the end of follow-up. Therefore, in this secondary study, the shared variables from previous studies were combined to deepen our knowledge of the evolution of breastfeeding in our environment. A combined sample of 314 participants was obtained. The selection criteria were identical in both studies. The inclusion criteria applied at the time of recruitment were being of legal age, having chosen to feed their newborns with exclusive breastfeeding, and voluntarily participating in the study. Multiple pregnancies were excluded, as well as those cases in which the mother and newborn were separated after childbirth due to maternal and/or neonatal health complications.

Furthermore, in both studies, women received the same support to promote and protect breastfeeding. Specifically, during the clinical postpartum period, nursing staff and midwives review the effectiveness of breastfeeding. After discharge, midwives (up to the first 6 weeks), pediatric nurses, and paediatricians assess the maintenance of breastfeeding. The follow-up and monitoring are established through the newborn programme, which includes a visit within 7 days postpartum by the midwife to both the mother and newborn, a 15-day check-up by the pediatric nurse and paediatrician, a 5–6 week check-up by the midwife, and follow-up visits at 2, 4, and 6 months by the paediatrician.

In both studies, breastfeeding was monitored through telephone calls to the participants during the first six months of the newborn’s life or until the definitive abandonment of breastfeeding occurred. If there was a change in the infants’ feeding, the main reason that had led to the change in the infants’ feeding was recorded, as well as the total weeks they were fed with each type of feeding (EBF: exclusive breastfeeding; SBF: supplemented breastfeeding; FF: formula feeding; total BF: sum of the weeks of EBF and SBF).

Those cases where breastfeeding monitoring began in the first month of the infant’s life, but it was impossible to contact the participants at any subsequent follow-up point were treated as censoured data.

### 2.3. Measurement

From the two cohorts from which the data were extracted, sociodemographic variables were recovered, such as the age of the participants and nationality (Spanish/other). Obstetric variables, such as parity (primiparous/multiparous) and variables related to breastfeeding (total weeks that EBF had been maintained, the SBF, and the main reason for change in the infant’s feeding: 1-Perception of insufficient milk production; 2-Weight gain less than recommended; 3-Breast problems resulting from breastfeeding; 4-Breast problems unrelated to breastfeeding; 5-Employment incorporation; 6-Any lack of breastfeeding support; 7-Another reason; 8-Introduction of Complementary Feeding).

### 2.4. Operational Definitions

According to previous studies [13,14], the following key situations can be distinguished.

- EBF cessation: a situation in which the baby stopped being fed with EBF to be fed with supplemented feeding
- Abandonment of BF: a situation in which the baby stopped receiving BF to be fed formula.

### 2.5. Statistical Analysis

Descriptive analysis used absolute and relative frequencies for qualitative variables and mean and standard deviation (SD) for quantitative variables. Bivariate analysis of the weeks until early EBF cessation and complete abandonment of breastfeeding was performed using the Mann–Whitney U test (dichotomous variables) and Kruskal–Wallis test (polychotomous variables). In addition, a detailed follow-up of the breastfeeding of each participant was carried out, reflecting the evolution of feeding during the first six months. In case a change in feeding occurred, the main reason for the change was detailed. In this way, the probability of feeding with each type of feeding (EBF, SBF, and FF) was calculated, as well as the probability that each reason for abandonment occurred depending on the follow-up time.

The formulas used to calculate the probability of feeding with each type of feeding at different follow-up times are as follows:

The prevalence (prev) of each feeding at each follow-up point was calculated as follows. In the numerator, we determined the type of feeding at that follow-up point. In the denominator, we found all the cases that, at the immediately previous follow-up point, were able to opt for the numerator type of feeding per 100 participants:

prev_EBF_ = (*n* _EBF i_/*n* _EBF previous_) × 100 (1)

prev_SBF_ = [*n* _SBF i_/(*n*_EBF previous_ + *n* _SBF previous_)] × 100 (2)

prev_FF_ = (*n* _FF i_/*n* _total_) × 100 (3)

The cumulative relative frequencies of feeding changes at each follow-up point were calculated as follows: the numerator contains the total number of infants who were fed by each of the feeding types at each follow-up point, while the denominator contains the total number of infants who, at the immediately preceding follow-up point, were fed by the original feeding type, per 100 participants.

For example, the calculation of the cumulative relative frequency (Fi) of those infants who, between the second and fourth months of follow-up, switch from exclusive breastfeeding to formula feeding is as follows: [(numerator: total number of infants who are fed by formula feeding at the fourth month of follow-up)/(denominator: total number of infants who are fed by exclusive breastfeeding at the second month of follow-up)] × 100:

Cumulative relative frequencies of changing from EBF to FF (Fi _1_) = (*n* _FF i/_*n* _EBF_ previous) × 100 (4)

Cumulative relative frequencies of changing from EBF to SBF (Fi _2_) = (*n* _SBF i/_*n* _EBF_ previous) × 100 (5)

Cumulative relative frequencies of changing from SBF to FF (Fi _3_) = (*n* _FF i/_*n* _SBF_ previous) × 100 (6)

The data were analysed with the SPSS v.28 software, and a statistical significance level of *p* < 0.05 was established.

## 3. Results

The sample consisted of 314 mother/baby dyads. The average age of the mothers was 33 years old (SD = 5), of whom 77.1% (*n* = 232) were of Spanish origin, and 51% (*n* = 160) were primiparous. By the first month, 10.2% (*n* = 32) of the participants had ceased exclusive breastfeeding (EBF). By the sixth month of follow-up, only 55.4% (*n* = 174) of the participants had continued EBF (Table 1).

**Figure 1:**
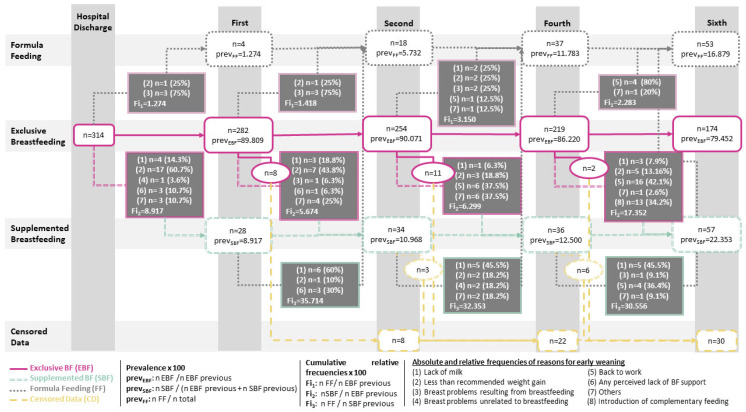
Evolution of breastfeeding throughout the first six months.

Figure 1 shows the evolution of breastfeeding over the first six months of the infants’ lives. Each monitoring point is shown in the columns of the figure. The rows show the different types of feeding and the absolute frequencies and prevalence (prev) of infants fed each type of feeding throughout the follow-up period. Between one monitoring point and the next, the flow of infants who change their feeding type is shown, making it possible to identify the absolute and relative frequencies of the main reason for the change in feeding and the respective accumulated relative frequencies (Fi).

Figure 1 shows that the prevalence of supplemental breastfeeding (SBF) and Formula Feeding (FF) increases as the months go by. In contrast, the prevalence of EBF decreases in the first month, remains until the second, and decreases until the sixth month. As for the cumulative relative frequencies of a particular feeding change occurring, it is observed that the change from EBF to SBF occurs to a greater extent between the fourth and sixth months (Fi_2_ = 17.352), as well as between hospital discharge and the first month (Fi_2_ = 8.917). The change from EBF to FF is the least common change throughout the entire follow-up, reaching its highest value between the second and fourth month (Fi_1_ = 3.150). In addition, approximately one-third of the infants fed with SBF end up with FF throughout the follow-up, although a slight decrease in this probability (Fi_3_) is observed as the months go by.

During the first two months of breastfeeding, the main reason for EBF cessation was the insufficient weight gain of the newborns (After discharge: (reason 2) *n* = 17, 60.7%; First month: (reason 2) *n* = 7, 43.8%). Among the participants who opted for SBF, the main reason for complete abandonment of breastfeeding throughout the entire follow-up was the perception of insufficient milk production (First month: (reason 1) *n* = 6, 60%; Second month: (reason 1) *n* = 5, 45.5%; Fourth month: (reason 1) *n* = 5, 45.5%). From the fourth month of breastfeeding, returning to work is the main reason for switching from EBF to SBF ((reason 5) *n* = 16, 42.1%), from EBF to FF ((reason 5) *n* = 4, 80%), as well as the second main reason for switching from SBF to FF ((reason 5) *n* = 4, 36.4%).

Table 2 shows the bivariate analysis of the average number of weeks that both EBF and total breastfeeding (EBF + SBF) are maintained. Significant differences were observed in terms of parity and reason for early weaning from breastfeeding. Specifically, multiparous women maintained, on average, almost 5 weeks more EBF (*p* < 0.001) and 3 weeks more breastfeeding in total (*p* < 0.001). However, no differences were observed between the country of maternal origin and duration of breastfeeding.

On the other hand, statistically significant differences are observed between the average number of weeks that EBF (*p* < 0.001), as well as total breastfeeding (*p* = 0.002), are maintained based on the reason for EBF cessation or abandonment of BF, respectively. It was possible to differentiate three groups of reasons based on the weeks that each of the breastfeeding types is maintained, with any lack of breastfeeding support and breast problems being the ones that determine a smaller number of weeks of EBF (2–5.4 weeks) and total breastfeeding (5.7–10 weeks); insufficient weight gain and the perception of insufficient milk production are the reasons that predominate in the central weeks of the follow-up (EBF: 6.2–8.7 weeks; total BF: 8.7–12.6 weeks), and returning to work is the late reason that prompts the early abandonment of both EBF and total breastfeeding.

## 4. Discussion

Our study highlights the importance of thoroughly understanding the patterns and factors that influence EBF and its transition to supplemental breastfeeding or total abandonment of breastfeeding. Despite the desire of Spanish mothers to breastfeed their babies, current data show a significant gap between maternal aspiration and the actual practice of EBF. The present study offers an invaluable opportunity to examine how breastfeeding evolves throughout the first six months of an infant’s life and what factors influence mothers’ infant feeding decisions. It is important to note that in our country, there is no official record of breastfeeding figures, which makes comparison difficult. The present study offers an invaluable opportunity to examine how breastfeeding evolves throughout the first six months of an infant’s life and what factors influence mothers’ infant feeding decisions through an innovative methodological approach.

According to Ballesta-Castillejos et al. [15], 97.5% of mothers in Spain wish to breastfeed their babies. However, the latest official data from 2017 reported that only 39% of infants under six months were exclusively breastfed (EBF) [7]. These results align with observations in the present study, where the likelihood of transitioning from EBF to supplemented breastfeeding (SBF) is higher at all follow-up points (5.674–17.352) than the probability of switching from EBF to formula feeding (FF) (1.418–3.150). These results support the idea that mothers have a genuine desire to breastfeed. Even when unable to exclusively breastfeed, they persist by supplementing breastfeeding as a final effort prior to ultimately ceasing it. However, once they start SBF, the likelihood of transitioning to FF increases significantly (30.556–35.714). These data highlight the importance of understanding the reasons behind supplementation or abandonment of breastfeeding.

One of the main factors negatively affecting the initiation and/or the continuation of breastfeeding is the lack of knowledge regarding the physiology of breastfeeding [10,16,17] and the creation of false expectations about the breastfeeding process. There is a misperception that breastfeeding is easy because of its inherent nature when, in fact, it requires learning and support [18]. The reported lack of knowledge negatively impacts mothers’ ability to handle physiological breastfeeding situations, such as growth spurts, reinforcing the suitability of educating mothers during the prenatal stage [11], theoretically and practically [19].

During growth spurts, the baby increases breast demand due to the need to boost milk production [20]. Additionally, babies may exhibit behaviours such as fussing at the breast, pulling away and seeking it constantly, and showing restlessness, among others. However, despite their aim to increase milk production, a lack of knowledge can lead mothers to misinterpret these signals as infant dissatisfaction [21,22,23] or a false perception of insufficient milk production [10,16,18,21,22,24]. The misperception of insufficient milk production can result in an absolute decrease in milk production if the infant is inadequately supplemented [23,24].

From hospital discharge until the first month of follow-up, the prevalence of EBF decreases by just over 10 percentage points. During this period, the first growth spurt typically occurs around the second–third week of life [20]. These data highlight the importance of knowledge of breastfeeding physiology in overcoming the first growth spurt while maintaining EBF. Mothers need to experience small successes with breastfeeding during the early postpartum period to develop self-efficacy in their ability to breastfeed and overcome breastfeeding challenges, as women’s self-efficacy is associated with the duration of EBF [25,26].

In the present study, the primary cause of EBF cessation during the first month is insufficient weight gain in newborns, consistent with previous studies [27]. Additionally, other predominant reasons include the perception of insufficient milk production and a perceived lack of breastfeeding support. Therefore, these results underscore the need for better postnatal support focused on empowering mothers in breastfeeding, as well as greater involvement of partners in breastfeeding to achieve proper EBF establishment. By safeguarding the initiation of EBF, inadequate weight gain in newborns can be prevented, thus avoiding early breastfeeding supplementation [23]. It is important to note that mothers who reported a lack of breastfeeding support as a reason for changing their babies’ feeding practices maintained EBF for only two and a half weeks and SBF for less than six weeks on average. These data are consistent with other studies showing that a positive attitude from the partner towards breastfeeding and adequate partner support perceived by the mother greatly influence the initiation [12,28,29,30] and duration of breastfeeding [28,29,30].

The probability of maintaining EBF in the second month is almost the same as that in the first month, while it decreases considerably by the fourth month. These results suggest that the second growth spurt, usually around six weeks of life [20], is easier to overcome than the third growth spurt, which occurs around three months. The reason why the second growth spurt is more accessible to overcome than the third one may be because, during the first two months, more postnatal visits are scheduled in Spain, both with midwives and paediatricians, facilitating better newborn weight control and resolution of doubts, provided that professionals committed to breastfeeding are available to avoid undue supplementation. However, after two months, scheduled consultations become less frequent, reducing passive support from health professionals to address the third growth spurt. Additionally, the third growth spurt presents other characteristics that may jeopardise maternal breastfeeding confidence, such as regulated milk production reducing the feeling of breast fullness, increased baby distractions during feeding, and slowed growth, among others.

In line with previous studies, as during the first month, the main reason for EBF cessation during the first two months is insufficient infant weight gain [10,27], while the primary reason for complete breastfeeding cessation during the first four months is the perception of insufficient milk [10]. Accordingly, when mothers cite insufficient infant weight gain as the primary reason for stopping breastfeeding, they maintain an average of six weeks of EBF and eight weeks of total breastfeeding, consistent with the average of 8.7 weeks observed in a previous study conducted in Spain [9]. In addition, when mothers perceive insufficient milk as the primary reason, they maintain an average of eight weeks of EBF and twelve weeks of total breastfeeding, which aligns with the average of 9.7 weeks found by Oribe et al. [9]. These data highlight that a lack of breastfeeding knowledge, coupled with insufficient commitment from health professionals, often leads to early breastfeeding supplementation, which, along with false perceptions and maternal insecurities, results in breastfeeding failure [23].

It is also important to mention that during the first two months, the primary reason for switching from EBF to complete breastfeeding cessation is breastfeeding-related breast problems. In fact, on average, women who reported breast problems during lactation only maintained EBF for five weeks and total breastfeeding for less than seven weeks. These results align with other previous studies conducted in Spain, which show that experiencing breast or breastfeeding problems constitutes the reason for the shortest breastfeeding duration [9,11]. These data reinforce the need to provide adequate breastfeeding support, especially during the early postpartum period, to ensure effective and properly established breastfeeding [27].

Additionally, from the fourth month of follow-up onwards, the primary reason for switching from EBF to SBF or FF is returning to work. These results are consistent with previous studies conducted in Spain [9,10,31], highlighting the need to advocate for social policies supporting breastfeeding that enable EBF until six months of age and facilitate the reconciliation of breastfeeding with work resumption [2,23].

### Limitations

Among the study’s limitations, the small sample size and the non-probabilistic sampling method used to recruit participants should be noted. Additionally, since this study is a secondary analysis of two previous studies conducted on the same population but at different times, the same variables were not considered in the respective data collection notebooks, resulting in a limited number of standard variables in both databases. Likewise, in both studies, any perception of lack of breastfeeding support was considered as a reason for abandonment, without differentiating whether the lack of support was from professionals, the family, or the couple. Likewise, it is challenging to compare institutional support for breastfeeding. Specifically, it is known that during the second data collection period, the participating hospitals were in phase 3D (Hospital General Universitario de Castellón and Hospital Lluís Alcanyís) and phase 1D (Hospital Universitario de La Ribera) of the Baby Friendly Hospital Initiative. However, the accreditation phase during the first data collection period is unknown. Understanding the implementation and impact of these evidence-based practices could provide valuable insights into how healthcare policies and interventions influence breastfeeding practices and outcomes.

However, despite these limitations, the results encourage future studies to deeply evaluate breastfeeding evolution with larger samples and greater methodological rigour, such as probabilistic sampling and expanding the number of variables and the possible reasons for early cessation of EBF and total breastfeeding.

## 5. Conclusions

The abandonment of breastfeeding is a multifactorial phenomenon. Even so, it seems that a specific factor generally predominates. Breast problems, as well as a lack of breastfeeding support, are observed as the reasons that promote the earliest abandonment of breastfeeding. In addition, early supplementation due to infants’ insufficient weight gain is a prevalent reason in our environment. Likewise, early supplementation usually leads to the perception of a lack of milk and prompts, which, within a few weeks, is the definitive abandonment of breastfeeding. From the fourth month of follow-up, returning to work seems to be the predominant reason for abandoning EBF.

Our study highlights the need to continue investing efforts to protect breastfeeding in our environment. Specifically, first, greater involvement in support for breastfeeding is needed from partners during the prenatal stage and early postpartum. Second, a more significant commitment from health providers is needed to empower mothers during the perinatal stage and prevent early and improper supplementation of breastfeeding. Third, it would be advisable to bet on social policies that make the maintenance of EBF viable until the infants are six months old. Finally, these findings not only inform on the need for adequate support and education for mothers during the breastfeeding process but also highlight the importance of social policies that support breastfeeding and facilitate its sustained practice over time, which has significant implications for child and maternal health in Spain.

## Figures and Tables

**Table 1:** Distribution of sociodemographic characteristics and feeding patterns during the first six months of life.

	*n*	%
Country of origin		
Spain	232	77.1
Other	68	22.9
Parity		
Primiparous	160	51
Multiparous	154	49
Feeding in the first month		
Exclusive Breastfeeding	282	89.8
Supplemented Breastfeeding	28	8.9
Formula Feeding	4	1.3
Feeding in the second month		
Exclusive Breastfeeding	254	80.9
Supplemented Breastfeeding	34	10.8
Formula Feeding	18	5.7
Censored Data	8	2.5
Feeding in the fourth month		
Exclusive Breastfeeding	219	69.7
Supplemented Breastfeeding	36	11.5
Formula Feeding	37	11.8
Censored Data	22	7
Feeding in the sixth month		
Exclusive Breastfeeding	174	55.4
Supplemented Breastfeeding	57	18.1
Formula Feeding	53	16.9
Censored Data	30	9.6

**Table 2:** Bivariate results regarding weeks of maintaining EBF and breastfeeding in total.

	Weeks Until Early EBF Cessation				Weeks Until Complete Abandonment of BF			
	*n*	*m*	*SD*	*p*	*n*	*m*	*SD*	*p*
Country of origin								
Spain	232	19.3	9.2	0.347 ^1^	232	22.1	7.2	0.724 ^1^
Other	68	18.2	9.4		68	21.7	7.6	
Parity								
Primiparous	160	16.8	9.9	<0.001 ^1^	160	20.5	8.3	<0.001 ^1^
Multiparous	154	21.4	7.8		154	23.7	5.7	
Reason for early EBF cessation/complete abandonment of BF, respectively								
Perception of lack of milk	11	8.7	6.6	<0.001 ^2^	18	12.6	7	0.002 ^2^
Less than recommended weight gain	32	6.2	6		7	8.7	4.3	
Breast problems resulting from BF	1	5.4	3.7		9	6.8	5.3	
Breast problems unrelated to BF	1	2	-		2	10	4.2	
Back to work	22	18.4	4.6		9	19.4	4.4	
Any lack of breastfeeding support	4	2.6	1.9		3	5.7	2.9	
Another reason	14	9.8	7.1		5	14.3	8.5	
Introduction of Complementary Feeding	13	21.2	0.6		-	-	-	

^1^ U de Mann–Whitney; ^2^ Kruskal–Wallis.

## Data Availability

The data presented in this study are available upon reasonable request and subject to privacy and ethical restrictions. For further information on how to submit a request for data access, please contact the corresponding author.

## Public Involvement Statement

No public involvement in any aspect of this research.

## Guidelines and Standards Statement

This manuscript was drafted against the Strengthening the Reporting of Observational Studies in Epidemiology (STROBE) Statement: guidelines for reporting cohort studies.
